# Physical Abilities in Low Back Pain Patients: A Cross-Sectional Study with Exploratory Comparison of Patient Subgroups

**DOI:** 10.3390/life11030226

**Published:** 2021-03-10

**Authors:** Nejc Šarabon, Nace Vreček, Christian Hofer, Stefan Löfler, Žiga Kozinc, Helmut Kern

**Affiliations:** 1 Faculty of Health Sciences, University of Primorska, 6310 Izola, Slovenia; nace.vrecek@gmail.com (N.V.); ziga.kozinc@fvz.upr.si (Ž.K.); 2 Laboratory for Motor Control and Motor Behaviour, S2P, Science to Practice Ltd., 1000 Ljubljana, Slovenia; 3 Human Health Department, InnoRenew CoE, 6310 Izola, Slovenia; 4 Ludwig Boltzmann Institute for Rehabilitation Research, 3100 St. Pölten, Austria; christian.hofer@rehabilitationresearch.eu (C.H.); stefan.loefler@rehabilitationresearch.eu (S.L.); 5 Institute for Physical Medicine, Physiko und Rheumatherapie, 3100 St. Pölten, Austria; helmut@kern-reha.at; 6 Andrej Marušič Institute, University of Primorska, 6000 Koper, Slovenia

**Keywords:** low back pain, strength, flexibility, mobility, radiated pain, discogenic pain, degeneration, biomechanics, function

## Abstract

An abundance of literature has investigated the association between low back pain (LBP) and physical ability or function. It has been shown that LBP patients display reduced range of motion, decreased balance ability, impaired proprioception, and lower strength compared to asymptomatic persons. The aim of this study was to investigate the differences between LBP patients and healthy controls in terms of several physical abilities. Based on the premised that different biomechanical and physiological causes and consequences could be related to different types of LBP, a secondary exploratory attempt of the study was to examine the differences between LBP subgroups based on the pain location (local or referred) or type of pathology (discogenic or degenerative) on the level of impairment of function and ability. Participants performed range of motion tests, trunk maximal voluntary contraction force tests, a sitting balance assessment, the timed up-and-go test, the chair rise test, and the trunk reposition error test. Compared to the control group, symptomatic patients on average showed 45.7% lower trunk extension (*p* < 0.001, η^2^ = 0.33) and 27.7 % lower trunk flexion force (*p* < 0.001, η^2^ = 0.37) during maximal voluntary contraction. LBP patients exhibited decreased sitting balance ability and lower scores in mobility tests (all *p* < 0.001). There were no differences between groups in Schober’s test and trunk repositioning error (*p* > 0.05). No differences were observed among the LBP subgroups. The exploratory analyses are limited by the sample size and uncertain validity of the diagnostic procedures within this study. Further studies with appropriate diagnostic procedures and perhaps a different subgrouping of the LBP patients are needed to elucidate if different types of LBP are related to altered biomechanics, physiology, and function.

## 1. Introduction

Lifetime prevalence of low back pain (LBP) is reported to be as high as 84% [1]. The total annual costs of LBP in the United States of America exceed 100 billion dollars, with two thirds of these costs being indirect due to lost wages and reduced productivity [2]. In the latest Global Burden of Disease Study, LBP was found to be the leading cause of disability around the world. It was estimated to be responsible for 58.2 million person-years lived with disability in 1990, increasing to 83 million in 2010 [3]. 

Several studies have investigated the association between LBP and physical ability, movement biomechanics, or function. It is important to note that causal relationships are often unclear in this context. Reduced range of motion (ROM) is well documented in LBP patients. For instance, lumbar LBP patients were shown to have reduced lumbar ROM compared to asymptomatic persons in a systematic review by Laird et al. [4]. Additionally, it was reported that the dynamic lumbar ROM was 10–15% smaller in chronic LBP patients compared to asymptomatic controls [5]. This reduction was negatively correlated with self-reported pain and kinesiophobia (defined as fear of pain with movement and assessed by Tampa Scale for Kinesiophobia) [5]. During squatting, LBP patients seem to increase their hip ROM in the sagittal plane, compensating for decreased lumbar flexion [6]. LBP patients were also shown to have reduced ROM during straight-leg rising test, which was caused by stretch intolerance rather than hamstring stiffness [7]. In lumbopelvic movements, the contribution of lumbar spine to the overall ROM is decreased in LBP patients [7]. Despite the fact that asymmetries in spinal ROM are common in general population, more pronounced asymmetries were observed in LBP patients [8].

Several studies have shown that LBP patients display decreased balance ability. Compared to healthy controls, LBP patients were reported to exhibit greater center of pressure (CoP) displacement [9,10] and velocity [11] during standing body sway tests and sitting balance tests with closed eyes [12]. It was indicated that this difference increases with the task difficulty [11]. In addition to increased body sway, lower scores on the star excursion balance test (which assessed the dynamic stability during reaching in different directions with the free leg during a single-leg stance) were also observed in the LBP group [10]. Finally, Ayhan et al. reported impaired voluntary control of body positioning, decreased movement control, delayed movement initiation, and a decreased ability to adapt to sudden surface changes [13]. Decreased balance in LBP patients appears to be associated with the presence of pain, but not its location and duration. In another study by Ruhe et al. [14], no association between pain severity and the magnitude of CoP excursions could be identified. 

An abundance of studies has shown decreased strength and muscular endurance in LBP patients. Inter-muscular imbalances in trunk muscle strength, particularly weaker trunk extensors, are strongly believed to be a significant risk factor for development of LBP, and LBP patients also display significantly lower trunk extensor electromyography (EMG) amplitudes [15,16,17]. Rossi et al. reported both trunk flexion and extension strength level to be reduced in the LBP group [18]. In addition, poor muscle endurance [18,19,20] and inappropriate endurance ratios [21] have been reported to be associated with LBP. Finally, LBP is associated with gluteus medius weakness [22] and muscle atrophy [19].

Furthermore, LBP is associated with impaired proprioception. Several studies investigated repositioning accuracy, which was found to be decreased in LBP patients for trunk flexion [23,24], but might be increased for trunk extension [24], perhaps due to the increased facet joint mechanoreceptors sensitivity. In contrast with these findings, Lee et al. showed no association between repositioning errors and LBP [25]. Two studies have investigated the effects of proprioceptive disruptions on repositioning accuracy in LBP patients and found that before the perturbations, they showed increased reposition error compared to controls, but when perturbations were added, they demonstrated no increase [26] or lower increase compared to healthy controls [27].

Altered biomechanics of movement, such as antalgic movement patterns and other protective strategies are also commonly associated with LBP [28]. Increased trunk stiffness during walking, particularly in the lumbo-pelvic area, is well documented [29,30]. However, while LBP patients are more stiff, Vogt et al. demonstrated higher stride-to-stride variability in lumbo-pelvic movement, resulting in a non-optimal gait pattern [31]. In uphill walking, LBP patients seem to have decreased stiffness of the trunk in the transversal and frontal plane [32]. In addition, Barzilay et al. reported decreased step length, gait velocity, and stride cadence in LBP participants compared to healthy controls [33]. Moreover, previous studies also documented poorer timed up-and-go test results in older patients with LBP, compared to controls who could have other reports of pain [34]. 

The primary aim of this study was to compare physical ability and function of LBP patients and healthy participants. While decreased physical ability was shown in LBP patients before, there is a paucity of research examining the differences between different types of pain (e.g., local and radiated) and pain mechanisms (discogenic or degenerative). Given that there are potentially many different underlying biomechanical or physiological mechanisms in LBP, our second aim was to conduct an exploratory preliminary analysis to investigate whether pain location or diagnosis regarding the pain mechanism (discogenic or degenerative) has influence on the level of impairment. Based on the current literature, we hypothesized that LBP patients would demonstrate restricted lumbar ROM, increased body sway during balance tasks, poorer trunk strength, altered gait pattern, lower gait velocity, and altered trunk proprioception compared to healthy controls. Due to the paucity of literature comparing different types of pain, we refrained from postulating a hypothesis as regards our second aim. 

## 2. Materials and Methods

### 2.1. Participants

The LBP group was comprised of chronic (duration of symptoms > 12 weeks) or chronic recurrent (>2 episodes in the last 6 months) [35] LBP patients who came to the Institute for Physical Medicine in Sankt Pölten and were diagnosed with LBP by a physician specialized in physical medicine and rehabilitation (physiatrist) using clinical examination, case history, and radiologic findings. Acute cases, regardless of the diagnosis, were not considered for this study. 

The patients were invited to voluntarily participate in the study, with the assurance that the performed testing would not affect their treatment. The LBP patients were further divided into groups by two criteria: pain location (local pain group—LP; local and radiated pain group—LRP) and diagnosis (intervertebral disc injury—DIS; degeneration or arthrosis—DEG). These classifications were based upon doctor’s clinical findings and radiologic examination. In some cases, patients could be classified based only on pain location, but not pain diagnosis. Therefore, the total sample sizes of symptomatic subgroups were not equal (i.e., LP + LRP was not equal to DIS + DEG; see Table 1 for all sample sizes). Radiated pain was defined by the pain from the lower back radiating to one or both limbs, regardless of the exact area. MRI images were performed in the case of suspected radiated pain. If the clinical examination and the MRI results were in agreement, we assumed radicular pain. If clinical examination did not agree with the MRI findings, we excluded the patient from the study (4.8% of cases). The pain was considered local when only the lumbar area showed tenderness, without any radiation. If the radiologic findings showed structural changes of the intervertebral discs (bulging of the discs, narrowing of intervertebral space, etc.) without any other accompanying structural bony or ligamentous changes, the pain was classified as discogenic. Degenerative pain was determined when there was a presence of structural changes other than changes to the discs, such as spinal stenosis, arthrosis, or presence of osteophytes (including the facet joints). Neuropathic pain was not included in the study. The control group (CG) consisted of asymptomatic participants, who were recruited through advertisements on social media and websites of the authors’ institutions. Basic information about groups of participants is presented in Table 1. Prior to the study, the participants were thoroughly informed about the procedures and were asked to sign an informed consent to participate. The measurement protocol was confirmed by the Ethics Committee in Niederösterreich, and the study was performed in consistency with the Oviedo Convention and the Declaration of Helsinki.

### 2.2. Pain Assessment

To investigate whether the subgroups of LBP patients differ regarding their current pain levels, they were asked to rate their pain levels on a 0–10 visual analogue scale (0 = no pain at all, 10 = worst pain imaginable). This was assessed as current pain (i.e., the pain on the day of testing) and also during trunk flexion and extension in order to assess movement-related pain. For this part, the participants were asked to slowly flex or extend the trunk towards the perceived end of their range of motion. The pain ratings were given immediately after they returned into the upright stance. The analysis revealed no statistically significant differences between the LBP subgroups (see Table 2 for details). The trends indicated that the LRP group could have higher pain levels, and that patients with discogenic pain might have higher pain levels associated with trunk flexion.

### 2.3. Body Mass and Body Composition Analysis (Bio-Electrical Impedance)

The participant was asked to step on the body composition monitor (BF511, Omron, Hoofddorp, Netherlands), which measured body mass, body fat (%), muscle tissue (%), visceral fat (%), and basal metabolic rate (kcal). We also calculated the body mass index (patient’s height and age were entered beforehand). Before the measurements, the participants performed a standardized warm-up, consisting of 9 dynamic exercises (~10 min).

### 2.4. Schober’s Test and Sacrum Inclination Angle Measurement

The participants were asked to take off their clothes (only for the duration of the tests in this section). They were notified beforehand to wear sports underwear. At the beginning of the test, the participants assumed an upright position. The examiner then located vertebrae L5/S1, TH12, and C7. The location of the L5/S1 vertebrae was determined at 3 cm below the horizontal line that connected the apexes of the iliac crest. The TH12 vertebrae were located at the level of costovertebral joint between the 12th rib and the spine. The C7 vertebrae were located as the most prominent vertebrae during neck flexion that were not moving when neck extension was performed. The examiner marked the positions of the vertebrae and measured the distances between L5/S1 and TH12, and between L5/S1 and C7 in an upright position. These distances were also used in the post-testing for more accurate results (at the POST visit, the examiner had to locate only the L5/S1 vertebrae; TH12 and C7 were located using the distances acquired in the pre-testing). The participant was asked to perform three maximal trunk flexions with knees fully extended. The examiner measured the distances between L5/S1–TH12 and L5/S1–C7 in a fully flexed trunk position. The result with the greatest distance between the vertebrae of the three repetitions was taken into further analysis. 

The sacrum inclination angle was measured with a digital inclinometer (vertical position = 0°). The lower part of the inclinometer was placed at height of vertebrae S3–S5 and was aligned with the spine. The patient was asked to perform three maximal trunk flexions with knees extended. The best result (i.e., the highest angle value) was taken into further analysis.

### 2.5. Trunk Repositioning Test

The participant was asked to take off his/her clothes and was given an eye patch to prevent visual feedback during the test. The participant performed three sets of pairs of repetitions of trunk flexion with the arms relaxed (hands not touching the legs). During the first repetition within each set, the examiner told the patient when to stop movement. The examiner then measured the angle of the trunk with a digital inclinometer placed on the TH12 vertebrae (using the mark from the Schober’s test as a reference). The participant was asked to remember the exact position of the trunk. The participant then returned to the neutral position and was asked to adopt the same flexed position of the trunk as in the first repetition. When the participant assumed what he/she thought was the same position, the examiner measured the angle of the trunk again. Average absolute error (absolute difference between guided position and assumed position) was calculated for further analysis.

### 2.6. Mobility Tests

The chair rising test (CRT) and the timed up-and-go (TUG) test were performed to assess mobility [36]. For the CRT, the participant was given instructions to rise and sit down again on a chair five times as fast as possible. The legs had to be fully extended when standing up and the buttocks had to touch the chair surface when sitting down. TUG test consisted of standing up from a chair, walking as fast as possible for three meters, turning around (a mark was marked on the floor), going back, and sitting down on the chair as fast as possible. The starting and the final positions were with the patient sitting on the chair and touching the backrest. For both tests, the time needed for test completion was measured manually with a stopwatch. Both tests were repeated 3 times, with a 30 s break in between the repetitions, and the best result (i.e., the shortest time needed for the execution) was taken into further analysis. If the participant appeared exhausted, a longer break between the repetitions was provided. 

### 2.7. Sitting Balance Test

**Figure 1:**
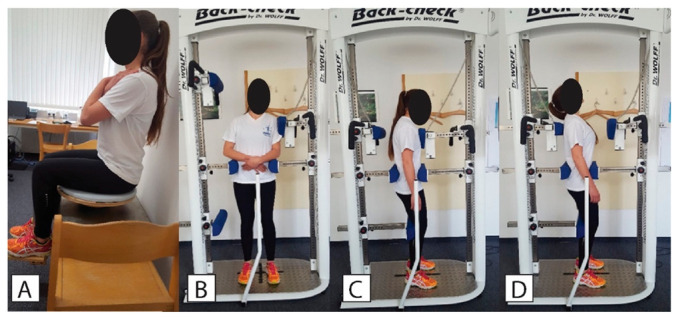
Sitting balance test (**A**), and measurements of strength for trunk lateral flexors (**B**), flexors (**C**), and extensors (**D**).

For assessment of sitting balance, a wobble board was placed on a force plate located on a seat/bed (Figure 1A). The participant was instructed to sit on the wobble board as still as possible without touching the plate or the wall. The hands were crossed in front of the chest and the participant was asked to look and concentrate on the dot, which was 2 cm in diameter and was positioned on the wall facing the participant, 2 m away and at eye level height. The feet were positioned on an extension that was attached to the wobble board, which prevented the upper limbs from being involved in balance control. Three measurements with 30 s breaks in between were performed. The acquisition time was set to 35 s, with the first 5 s not being taken into further analysis. The examiner was standing by the patient at all times in case the patient would lose control. The data were recorded and automatically processed in Fitro Sway software (Version 3.1., Fitronic s.r.o., Bratislava, Slovakia). The main outcome measures were velocity, amplitude, and frequency of the CoP movement, examined in both antero-posterior (AP) and medio-lateral (ML) directions.

### 2.8. Trunk Strength

The dynamometer used in this study was BACK-CHECK® 607, (Dr. WOLFF Sports & Prevention GmbH, Arnsberg, Germany), which allows testing trunk strength (Figure 1B–D) in the frontal and sagittal plane while providing firm fixation at the pelvic level to ensure that the lower limbs do not contribute to the recorded values. This dynamometer is considered as a gold standard device and has been used in previous studies [37,38]. Similar procedures have also been proven reliable with less complex trunk dynamometers [39]. For the assessment of the lateral flexors of the trunk, the participant was positioned in the middle of the dynamometer with the shoulders positioned against the lateral force meter and arms crossed in front of the abdomen. The patient was fixed using straps, with the height of the fixators aligned with the patient’s crista iliaca on both sides. Horizontal sagittal fixators were adjusted so that the patient was positioned in the middle of the dynamometer. The height of the upper edge of the sagittal force sensor was aligned with the patient’s shoulder and the horizontal setting was adjusted so that the patient was slightly touching the fixator pad in an upright position. 

For the assessment of flexors and extensors of the trunk, the patient was positioned in the middle of the dynamometer with the chest facing towards the flexion force meter and arms hanging beside the body. The height of the leg fixator was aligned with the patient’s knee height. The horizontal setting was adjusted to prevent knee flexion. The height of the anterior and posterior hip and pelvis fixators was aligned with the patient’s spina iliaca anterior superior and with spina iliaca posterior superior, respectively. Horizontal adjustments of the posterior fixators were made so that the patient was positioned in the middle of the dynamometer. The height of the flexion force sensor was aligned with the patient’s sternum. The height of extension force sensor was aligned with the spine of the scapula. The horizontal settings were adjusted so that the patient was slightly touching the force meter pad in an upright position.

All measurements were performed in isometric conditions. The patient was asked to perform three repetitions of maximal trunk lateral flexion left/right, trunk extension, and trunk flexion, respectively. The best result (highest value) was taken into further analysis). The breaks between repetitions lasted approximately 10 s and were extended if the patient was showing signs of exhaustion. The examiner was observing for any irregularities in the execution (e.g., hitting the force meter and thus creating a thrust, which is not a realistic indicator of muscle strength, or rotating the trunk and lifting feet off the ground). 

### 2.9. Statistical Analysis

Statistical analysis was performed in SPSS Software (Version 20.0, IBM, Armonk, NY, USA). Descriptive statistics (mean ± standard deviation) were calculated for all parameters. The normality of distribution was assessed with the Shapiro–Wilk test. For the outcomes that violated the normality criteria, we used logarithmic, quadratic and Box–Cox transformations. Two separate analyses were conducted based on the symptomatic group division criteria (based on pain location and based on diagnosis). We used the one-way analysis of variance to test the differences between the groups. Effect sizes were expressed with eta-squared (η^2^) and interpreted as small (~0.01), medium (~0.06), and large (~0.14). When normality of distribution could not be met with performing transformations, the Kruskall–Wallis test was used. The independent sample t-test or Mann–Whitney U test were used for pairwise comparisons. The threshold for statistical significance was set at *p* < 0.05 for all comparisons.

## 3. Results

**Figure 2:**
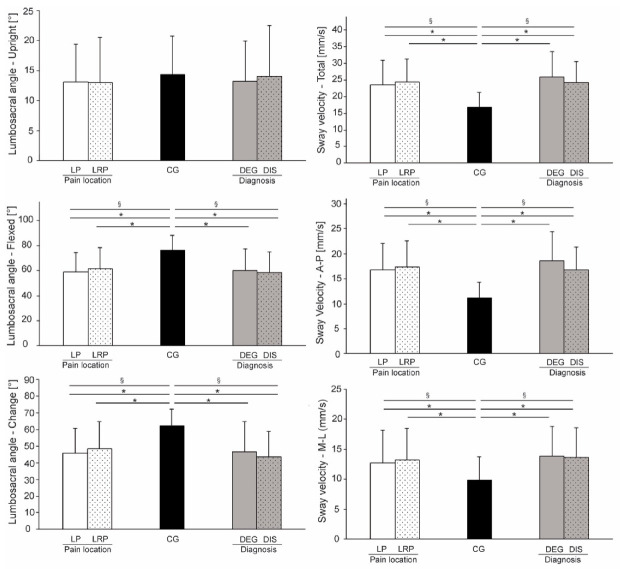
Differences between groups regarding sacrum inclination (left panel) and center of pressure (CoP) velocity during sitting balance test (right panel). § denotes a statistically significant main effect of one-way analysis of variance, and * indicates statistically significant post-hoc comparisons (*p* < 0.005).

No significant differences were found between LBP groups compared to the CG for the Schober test (*p* > 0.05). Mean sacrum inclination in upright stance was not statistically significant (*p* = 0.4–0.6) relative to the CG, whereas mean sacrum inclination in bent position (F_2, 215_ = 17.965, *p* < 0.001, η^2^ = 0.378 and F_2, 104_ = 14.953, *p* < 0.001, η^2^ = 0.473, with participants grouped by pain location and diagnosis, respectively) showed significant differences relative to the CG. Statistically significant differences for the change of sacrum inclination were observed relative to the CG (F_2, 215_ = 17.293, *p* < 0.001, η^2^ = 0.372 and F_2, 104_ = 16.218, *p* < 0.001, η^2^ = 0.488, for pain location and diagnosis, respectively). Post hoc pairwise tests did not reveal any statistically significant differences between LBP subgroups (LP compared to LRP and DIS compared to DEG) (*p* = 0.2–0.9). Detailed results regarding sacrum inclination are provided on Figure 2 (left panel).

The trunk reposition error test showed no significant differences between the compared groups (*p* = 0.116, η^2^ = 0.139, and *p* = 0.794, η^2^ = 0.065, for pain and pathology type, respectively). Statistically significant were differences between groups for mean CRT scores (*χ*^2^_(2)_ = 62.587, *p* < 0.001, η^2^ = 0.275 and *χ*^2^ (2) = 57.202, *p* < 0.001, η^2^ = 0.506, for pain location and diagnosis, respectively). Post hoc pairwise tests did not reveal any statistically significant differences between LBP subgroups (*p* = 0.51 for pain location and *p* = 0.656 for diagnosis). Mean TUG scores were significantly better in the CG compared to the LBP subgroups (*χ*^2^_(2)_ = 22.574, *p* < 0.001, η^2^ = 0.092 and *χ*^2^ (2) = 32.639, *p* < 0.001, η^2^ = 0.266, for pain location and diagnosis, respectively). Post hoc pairwise tests did not reveal any statistically significant differences between LBP subgroups (*p* = 0.165 for pain location and *p* = 0.63 for diagnosis).

In the sitting balance assessment, total CoP velocity was significantly smaller in the CG compared to the LBP subgroups (F_2, 131_ = 17.825, *p* < 0.001, η^2^ = 0.463 and F_2, 64_ = 20.965, *p* < 0.001, η^2^ = 0.629, for pain location and diagnosis, respectively). The CG also showed statistically significant smaller values for AP CoP velocity (F_2, 131_ = 21.082, *p* < 0.001, η^2^ = 0.493 and F_2, 64_ = 23.982, *p* < 0.001, η^2^ = 0.655, for pain location and diagnosis, respectively) as well as for ML CoP velocity (F_2, 131_ = 5.271, *p* = 0.006, η^2^ = 0.273 and F _2, 64_ = 6.961, *p* = 0.002, η^2^ = 0.433, for pain location and diagnosis, respectively). Detailed results regarding CoP velocity are provided in Figure 2 (right panel). Both the AP and ML CoP amplitudes were significantly different when comparing the CG to the LBP subgroups (AP direction; F_2, 131_ = 19.959, *p* < 0.001, η^2^ = 0.483 and F _2, 64_ = 18.465, *p* < 0.001, η^2^ = 0.605, for pain location and diagnosis, respectively; ML direction: F_2, 131_ = 11.572, *p* < 0.001, η^2^ = 0.387 and F_2, 64_ = 15.456, *p* < 0.001, η^2^ = 0.571, for pain location and diagnosis, respectively).

In both directions, the CoP frequency derived from peak excursion was significantly smaller in the LBP groups (AP direction: F_2, 131_ = 3.933, *p* = 0.022, η^2^ = 0.238; ML direction: *χ*^2^_(2)_ = 11.789, *p* < 0.001, η^2^ = 0.092) for pain location subgroups, as well as for diagnosis subgroups (AP direction: F_2, 64_ = 3.646, *p* = 0.032, η^2^ = 0.32; ML direction; *χ*^2^_(2)_ = 32.639, *p* = 0.006, η^2^ = 0.13) when compared to the CG. In contrast, the CoP frequency derived from power spectrum was not different between groups (*p* = 0.1–0.5). However, post hoc pairwise tests showed statistical difference in AP direction (*t* = −2.036, *p* = 0.048, ES = 0.6) between the CG and DEG subgroup. Other post hoc tests did not reveal any statistically significant differences between subgroups (*p* = 0.196–0.217). 

**Figure 3:**
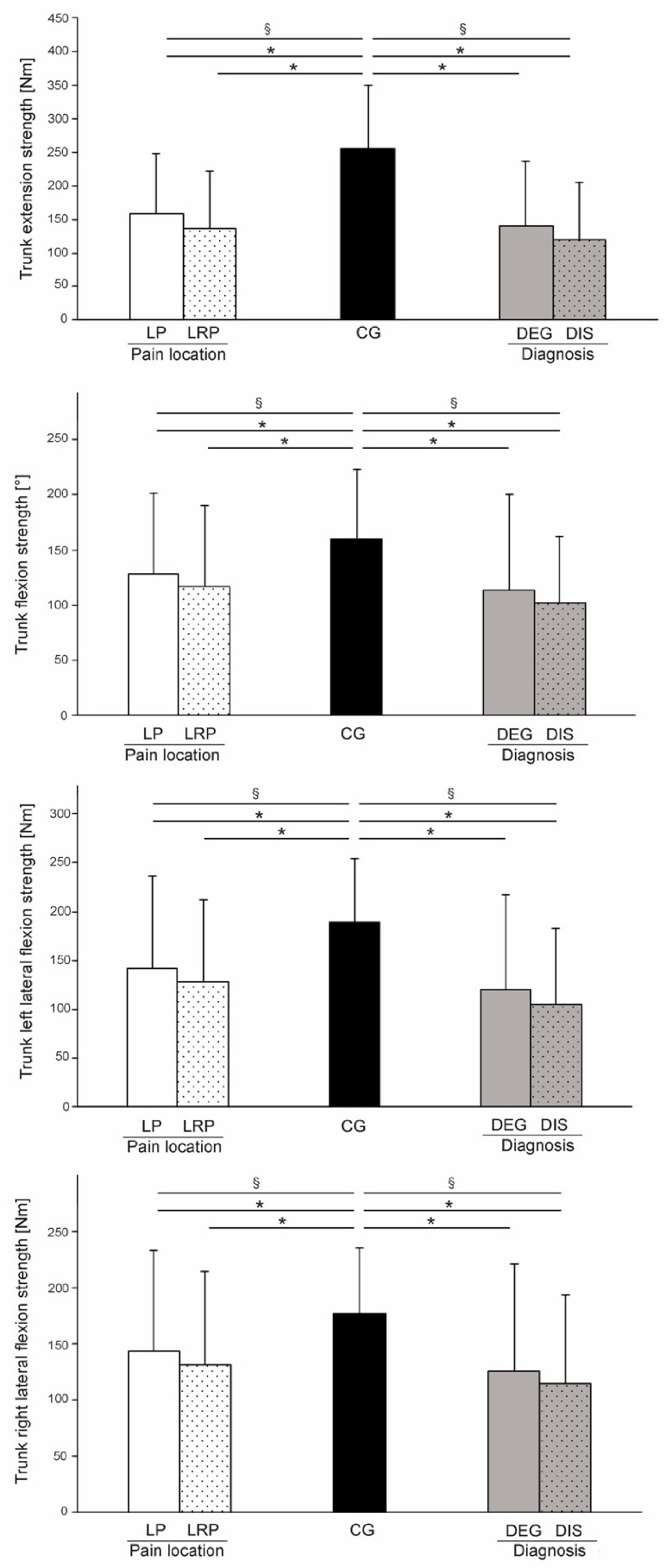
Differences between groups regarding trunk extension (upper), trunk flexion (middle), and trunk lateral flexion (lower) strength. Note that only left lateral flexion is shown, as both sides showed virtually identical results. § denotes a statistically significant main effect of one-way analysis of variance, and * indicates statistically significant post-hoc comparisons (*p* < 0.005).

Mean trunk extension strength (Figure 3) for the CG was significantly higher in comparison to the subgroups (F_2, 223_ = 21.595, *p* < 0.001, η^2^ = 0.327 and F_2, 111_= 20.658, *p* < 0.001, η^2^ = 0.521, for pain location and diagnosis, respectively). Similarly, the CG showed statistically significant higher mean values for trunk flexion strength compared to the two groups (*χ*^2^ (2) = 11.201, *p* < 0,001, η^2^ = 0.041 and F_2, 111_ = 8.767, *p* < 0.001, η^2^ = 0.369, for pain location and diagnosis, respectively). Lateral flexion strength in both left and right direction showed statistically significant differences when comparing the CG to the subgroups pain location (*χ*^2^ (2) =11.896 and 19.176, *p* < 0.001, η^2^ = 0.077 and 0.044) and pathology type (F_2, 112_ = 15.705 and F_2, 111_ = 8.474, *p* < 0.01, η^2^ = 0.468 and 0.364). Post hoc pairwise tests did not reveal any statistically significant differences between the LBP subgroups (*p* = 0.06–0.4 for pain location and *p* = 0.4–0.9 for diagnosis).

## 4. Discussion

The aim of this study was to compare the physical abilities of LBP patients with different types of LBP (based on location and diagnosis) and healthy participants by assessing several functional and biomechanical aspects. Based on the previous investigations, we hypothesized that LBP patients will demonstrate restricted lumbar ROM, increased body sway during balance tasks, poorer trunk strength, altered gait pattern, lower gait velocity, and altered trunk proprioception compared to healthy controls. LBP patients indeed exhibited decreased abilities in terms of trunk strength, sitting balance, and mobility. On the other hand, their performance in terms of trunk repositioning and Schober’s test was similar to healthy controls. We found no evidence to support the differences between LBP subgroups based on pain location or diagnosis. This can mean that a) the abilities that we explored are impaired to a similar extent in different types of LBP, or b) the classification of LBP into subgroups was not optimal in our study and should be revised and refined.

No statistically significant differences were observed between the CG and LBP patients in spinal ROM, although a tendency of hypo-flexibility in lumbar part and hyper-flexibility in thoracic part of the spine can be seen in symptomatic patients compared to the CG, similar to conclusions of Laird et al. [7]. In contrast to our findings, they found statistically significant differences between the CG and LBP patients regarding pelvic mobility (change of sacrum angle between upright and fully flexed position). Reduced lumbar spine flexibility could have resulted from increased stiffness or co-activation of lower back muscles as a protective mechanism against exacerbating of the symptoms. Difference in the methods of testing flexibility (digital inclinometer for pelvic mobility testing versus the measurement tape for spinal ROM) could be the main reason for observed differences in the results.

The literature regarding trunk proprioception or trunk repositioning error during forward bending in LBP patients is contradictory. Newcomer et al. [24] performed a study to investigate the differences between LBP patients and healthy controls regarding trunk repositioning error during 30%, 60%, and 90% of maximal forward trunk flexion and extension. They reported that LBP patients showed higher errors in flexion but lower errors in extension. The authors concluded that increase flexion errors imply that the proprioception is deteriorated to some degree. They also suggested that the contrast findings for extensions could be explained by increased sensory inflow, possibly due to an increased activation of mechanoreceptors in facet joints. In their next study [36], the authors performed a similar testing protocol with fixation of lower extremities and pelvis, and thereby a limited sensory feedback from distal receptors. In this study, no statistically significant differences were found between the two groups for any of the movements. In the present study, we have failed to show statistically significant differences between the CG and LBP patients. One of the reasons for the absence of differences could be, as previously suggested [40], a poor choice of testing position. The sensory feedback from distal receptors could be a significant factor, explaining the absence of differences. Another reason for the lack of differences could be linked to pain or pain intensity in the reference positions. Mechanisms of pain irritation can be chemical or mechanical, so the LBP patients may have used pain intensity as a feedback of position, thereby reducing the proprioceptive component of the testing. Reference positions should have been used in smaller ROMs so the effect of pain is minimized.

The differences between groups regarding the CRT and TUG results indicate an impaired function of lower extremities and reduced mobility in LBP patients, since the observed differences were large (CG: 5.4 ± 1.5 s, LBP groups: 10.4–3.2 ± 5.3–10.2 s) One of the possible explanations for observed differences is that LBP patients adopt behavioural patterns, such as decreased physical activity and deconditioning of lower extremities. Moreover, discomfort was seen in some of the symptomatic patients during the testing. Although this was not assessed in this study, we could assume these patients did not perform the tests with maximal effort. Future studies should consider monitoring the perceived level of effort and discomfort during the execution of the tasks to provide more information regarding the underlying mechanisms of the observed differences related to the CRT and TUG. Notably, Rodrigues et al. reported no statistically significant differences in the CRT results between healthy and symptomatic patients, who were matched by age, gender and by the amount of weekly physical activity [41]. According to the average body mass of compared groups in our study, we could speculate that the LBP patients were physically less active than the CG, but we cannot know whether this phenomenon was established before the onset of LBP, nor do we know whether that was the cause for lower performance in the CRT and TUG. Furthermore, previous studies have demonstrated statistically significantly slower walking speed compared to healthy controls [42]. Moreover, it was shown that a reduction in severity of LBP is associated with a concomitant improvement in gait velocity, step length, and gait cadence [33]. The question remains whether reduced mobility in LBP is mostly due to the behavioural changes or due to the direct effects on gait and overall function.

LBP patients showed greater CoP velocity and amplitude of displacement during the sitting balance task, thereby exhibiting poorer sitting balance or postural control of the lumbar part of the spine and trunk stability compared to the CG. The seat with a semi-circular base was designed with a support for both feet and primarily challenged lumbar or core stability with minimal contribution of lower extremities. LBP patients were shown to have greater trunk muscle co-contraction and greater trunk stiffness during balance tasks compared to healthy controls [43]. Moreover, LPB patients showed delayed reflex muscle responses after an unexpected applied mechanical perturbation [44]. The co-contraction could serve as a protective mechanism with the purpose of restricting spine motion and thus prevent further exacerbation of the condition. Altered muscle activation patterns and responses could also be any of the causes of non-specific LBP [45,46,47]. Van Dieën et al. have attributed altered muscle activation patterns to compensatory mechanisms that emerge to increase the stability of the spine [48]. It was also proposed that proprioception deficits in lumbar part of the spine are a major cause for poorer lumbar postural control [44].

The studies to date that have investigated the differences in trunk strength in LBP patients have reported reduced values compared to healthy controls [49]. In our study, we have observed statistically significant differences between the CG and LBP patients. Compared to the CG, LBP patients showed on average 45.7% lower trunk extension strength, 27.7% lower trunk flexion strength, and 34.4% and 27.3% lower trunk lateral flexion strength in left and right direction, respectively. Chiou et al. [49] reported reduced trunk extensor muscles activation levels during maximal voluntary contraction testing in isometric conditions. Reduced muscle activation in LBP patients is believed to be the consequence of reduced neural drive. For a better and more accurate interpretation of results achieved in strength testing, it would be more convenient to calculate muscle torque normalized to body mass (N/kg) because there was a large variation of patient body mass and body mass index (CG: 24.6 ± 2.9; LBP groups: 28.4–29.2 ± 4.2–5.7). Smaller trunk torque achieved by LBP patients could be a result of motion related pain, which prevented them from performing the test maximally. In the presence of pain or even fear of pain, the activity of the central nerve system is reduced to prevent the aggravation of pain [50]. 

Our results showed no differences in terms of any of the outcomes among groups of LBP patients. This is, to the best of our knowledge, the first study to explore different types of LBP and their effect on physical abilities and function. Due to the lack of previous studies, it is hard to suggest the reason for absence of differences. It could be expected, for instance, that radiated pain and discogenic pain could contribute to more pronounced reductions in performance in the test that involves the flexion of the trunk, due to the possible neural involvement [51] or flexion-related pain [52]. Moreover, the pain that radiates towards lower extremities was shown to alter gait characteristics [53,54], which could impair performance in the CRT and TUG. However, no indications for the effects of pain location or diagnosis were observed in this study. Therefore, more studies are needed to determine which tests are useful to discriminate patients with different types of LBP. Other types or mechanisms of LBP, such as fasciae [55] or other subpopulations of LBP patients, such as athletes [56], should also be considered in the future. 

A major limitation of this study should be acknowledged. Namely, it remains unknown whether the observed differences between the CG and LBP groups can be attributed solely to the presence of LBP, or whether some amount of difference had existed even before the LBP occurred. To clarify this uncertainty, a prospective study with a large sample size is needed in the future. A further limitation is the discrepancy between the CG and LBP groups regarding body composition. Consequently, the lower performance of LBP patients could be in part due to the higher body mass and not due to LBP. The tests of spinal flexibility/mobility and reposition error are possibly prone to examiner errors. Instrumented tests of spinal flexibility (such as radiographic assessments) and repositioning ability could lead to different results, and therefore the outcomes reported in the present study need to be viewed with caution.

Finally, a major limitation pertaining to our second exploratory aim is in the fact that the origins and/or mechanisms of pain could not be determined with absolute certainty. Despite the use of a combination of clinical examination and MRI investigation, it cannot be determined whether the structural changes observed were the reason (or at least the only reason) for the pain in the patients. Therefore, other mechanisms or sources of pain might have interfered with our analyses. The subgrouping of the patients in this study is clearly based on criterions with uncertain validity, and therefore it remains unknown whether the lack of observed differences reflect the true characteristics of LBP subgroups or whether they occurred due to non-optimal diagnostics. Other subgroup systems based on even more reliable diagnostic procedures should be used in the future studies to answer the questions regarding the influence of LBP type on physical abilities and function. Moreover, we encourage future researchers to assess, report, and consider in their statistical analyses all potential confounding factors, such as the level of kinesiophobia, which could influence the outcomes of the physical abilities tests.

## 5. Conclusions

The participants with LBP have demonstrated lower performance in terms of trunk strength, sitting balance, and mobility. On the other hand, no differences were observed compared to healthy controls in view of trunk repositioning errors and Schober’s test. We found no indications that the location or type of pain in LBP patients influenced any of the outcomes. It appears that none of the assessment approaches that have been used in this study are sensitive to specific types of LBP. Therefore, subsequent research will be needed to demonstrate potential biomechanical, physiological, or functional differences among different types of LBP.

## Figures and Tables

**Table 1:** Descriptive data for the groups of participants.

	All (n)	Female (n)	Age (years)	Body Height (cm)	Body Mass Index (kg/m^2^)
CG	37	15	49.4 ± 13.7	173.7 ± 7.6	24.6 ± 2.9
LBP-LP	122	49	49.0 ± 16.1	170.6 ± 8.8	29.2 ± 5.7
LBP-LRP	69	31	51.2 ± 12.6	170.5 ± 8.5	28.4 ± 4.2
LBP-DIS	42	20	56.5 ± 14.0	169.4 ± 8.1	28.7 ± 4.8
LBP-DEG	40	21	48.7 ± 12.2	171.0 ± 9.2	28.8 ± 5.2

CG—control group; LBP—low back pain; LP—local pain group; LRP—local and radiated pain group; DIS—intervertebral disc injury group; DEG—degeneration or arthrosis group.

**Table 2:** Outcomes regarding the pain across the patients’ subgroups.

Subgroup/Outcome	Local Pain		Radiated Pain		Difference	
	Mean	SD	Mean	SD	*t*	*p*
Pain-General	3.56	1.918	4.24	2.513	−1.389	0.171
Pain-Flexion	3.86	2.043	4.45	2.751	−1.120	0.268
Pain-Extension	4.41	2.457	5.02	2.437	−1.159	0.249
Subgroup/Outcome	Discogenic Pain		Degenerative Pain		Difference	
	Mean	SD	Mean	SD	*t*	*p*
Pain-General	4.07	2.433	4.12	2.303	0.126	0.900
Pain-Flexion	4.68	2.816	3.45	2.074	1.983	0.053
Pain-Extension	4.93	2.418	4.57	2.68	0.577	0.566

SD—standard deviation; *t*—*t*-test statistics; *p*—statistical significance.

## Data Availability

The data is available upon request to corresponding author’s email.
